# Changes in cardiovascular-health blood biomarkers in response to exercise intervention among older adults with cognitive frailty: A scoping review

**DOI:** 10.3389/fphys.2023.1077078

**Published:** 2023-02-15

**Authors:** Azianah Ibrahim, Arimi Fitri Mat Ludin, Devinder Kaur Ajit Singh, Nor Fadilah Rajab, Suzana Shahar

**Affiliations:** Centre for Healthy Ageing and Wellness (HCARE), Faculty of Health Sciences, Universiti Kebangsaan, Kuala Lumpur, Malaysia

**Keywords:** cognition, frailty, exercise, ageing, biomarkers

## Abstract

**Introduction:** Cardiovascular health contributes significantly to the incidence of cognitive impairment. Prior to conducting exercise-related intervention, it is crucial to explore cardiovascular health blood parameters that have been commonly used as guidance for the purpose of monitoring. Information on the effectiveness of exercise on cardiovascular-related biomarkers is lacking, especially among older adults with cognitive frailty. Therefore, we aimed to review existing evidence on cardiovascular-related blood parameters and their changes following exercise intervention among older adults with cognitive frailty.

**Methods:** A systematic search was conducted on PubMed, Cochrane, and Scopus databases. Related studies involving only human and full text in either English or Malay language were selected. Types of impairment were limited to cognitive impairment, frailty, and cognitive frailty. Studies were restricted to randomized controlled trial and clinical trial design studies. For charting purposes, all variables were extracted and tabulated. Trends in types of parameters studied were explored.

**Results:** A total of 607 articles were screened, and the final 16 were included in this review. Four cardiovascular-related blood parameter categories were extracted: inflammatory, glucose homeostasis, lipid profile, and hemostatic biomarkers. The common parameters monitored were IGF-1 and HbA1c, glucose, and insulin sensitivity in some studies. Out of the nine studies on inflammatory biomarkers, exercise interventions showed a reduction in pro-inflammatory markers, namely, IL-6, TNF-α, IL-15, leptin, and C-reactive protein and an increase in anti-inflammatory markers, namely, IFN-γ and IL-10. Similarly, in all eight studies, glucose homeostasis-related biomarkers had improved with exercise intervention. The lipid profile was tested in five studies, with four studies showing improvements with exercise intervention *via* a decrease in total cholesterol, triglycerides, and low-density lipoprotein and an increase in high-density lipoprotein. A decrease in pro-inflammatory biomarkers and an increase in anti-inflammatory biomarkers were demonstrated with multicomponent exercise, including aerobic exercise in six studies and aerobic exercise on its own in the remaining two studies. Meanwhile, four out of six studies that yielded improvements in glucose homeostasis biomarkers involved only aerobic exercise and the remaining two studies involved multicomponent with aerobic exercise.

**Conclusion:** The most consistent blood parameters studied were glucose homeostasis and inflammatory biomarkers. These parameters have been shown to improve with multicomponent exercise programs, particularly with the inclusion of aerobic exercise.

## 1 Introduction

Cognitive frailty is a significant predictor of dementia (Bu et al., 2021). This is signified by cognitive impairment and physical frailty, according to the definition provided by the International Academy on Nutrition and Aging and the International Association of Gerontology and Geriatrics (Kelaiditi et al., 2013). Cardiovascular health (CVH) has been consistently identified as a dementia risk factor, and most importantly, it is one of the modifiable risk factor (Livingston et al., 2020; Wu et al., 2022). CVH is the health of heart and blood indicated by smoking, diet, physical activity, body mass index, blood pressure, total cholesterol, and fasting glucose and blood parameter and is a consistent and sensitive outcome measure for changes in CVH (Lloyd-Jones et al., 2010).

Most of the studies have utilized lipid profile (total serum cholesterol and triglycerides), glucose homeostasis (glucose and hemoglobin A1c), and hemostatic markers (hemoglobin and homocysteine) to assess CVH as a risk factor among older adults with dementia or cognitive impairment (Samieri et al., 2018; Peters et al., 2019). On the effectiveness of intervention, the review by Lin et al. (2015) on unspecified adults reported that exercise improved lipid profiles, glucose metabolism, and anti-inflammatory markers. On the contrary, the effectiveness of exercise on CVH-related blood parameters among 1,567 healthy older adults with three-arm intervention using high-density lipoprotein (HDL), low-density lipoprotein (LDL), total cholesterol (TC), triglycerides (TG), hemoglobin A1c (HbA1c), and glucose did not show any changes or improvements (Letnes et al., 2022). Almost similar systematic reviews dated until March 2014 have been conducted on general adults without age limit and impairment. There is a lack of information on the effectiveness of exercise on cardiovascular-related biomarkers, particularly among older adults with cognitive frailty (CF).

Therefore, this review aimed to scope the information on cardiovascular-related blood parameters among older adults with CF in response to exercise intervention. This will aid in the identification and choice of cardiovascular-related blood parameters to be assessed as changes in CVH with exercise intervention. This syndrome, CF, is a probable precursor to negative health-related consequences, namely, falling, injuries, and disability among older adults (Rivan et al., 2021; Richter et al., 2022).

## 2 Methods

This scoping review was prepared based on the Arksey and Malley (2007) framework. Our protocol followed the Preferred Reporting Items for Systematic Reviews and Meta-analysis protocol for scoping reviews (PRISMA-ScR) (Tricco et al., 2018). All methods were uploaded on the Open Science Framework (Ibrahim, 2020).

### 2.1 Stage 1: Identify the research question

The research questions addressed in this study were: what are the cardiovascular-health biomarkers that have been studied among older adults with cognitive frailty who underwent exercise intervention? Second, which type of exercise intervention yields changes in cardiovascular-health biomarkers upon intervention among older adults with cognitive frailty? Both single exercise interventions and multicomponent interventions were included. Due to the availability of the limited number of studies related to this area, older adults with cognitive frailty and cognitive impairment or a frail population was included in this review.

### 2.2 Stage 2: Identify relevant studies

We used the mnemonic PCC: population, context, and concept in Table 1 as a guide for identifying the relevant studies, particularly those focused on cardiovascular-health blood biomarkers among older adults with cognitive frailty who undertook exercise intervention. PubMed, Cochrane, and Scopus were used to search for peer-reviewed studies published in English and Malay.

**TABLE 1:** PCC mnemonic.

Population	Older person with cognitive impairment, frailty, or cognitive frailty
**C**oncept	Cardiovascular biomarker
**C**ontext	Exercise intervention

A combination of keywords and MeSH terms were used based on the theme of the research questions. The following keywords were used:

1. Exercise OR “physical activity” OR “physical exercise*” OR aerobic OR endurance OR Strength* OR “exercise training*” OR “strength training.”
2. “Older adult” OR old* OR elderly OR geriatric.
3. “Cognitive frail*” OR frail* OR “cognitive impairment” OR “mild cognitive impairment.”
4. Biomarkers OR biochemical OR “marker” OR “biological marker” OR “clinical marker” OR laboratory OR “bio-indicator.”
5. Randomized control* trial OR randomized controlled trial OR controlled clinical trial OR random allocation OR intervention studies.

### 2.3 Stage 3: Study selection

The inclusion criteria were full peer-reviewed, journal articles in English or Malay language, an age limit of 60 and above, and a randomized controlled trial study design in which participants with cognitive impairment, frailty, or cognitive frailty were assigned to at least one group of participants for exercise and in which cardiovascular-related blood biomarkers among older adults were examined. We excluded articles based on one or more of the following reasons: study subjects were not human, pediatric population, and duplicate and/or unrelated study topic. To establish the protocol for determining eligibility, two content experts AI and AFML reviewed the title and abstracts. Once reviewers established that the selected articles met the eligibility criteria and full consensus, the criteria for article identification and inclusion were applied. The Rayyan platform was used for screening purposes (Ouzzani et al., 2017).

### 2.4 Stage 4: Charting the data

The following information was extracted from every eligible record: author(s), year of publication, age, sex, body mass index, duration of exercise intervention, details of exercise, type of biomarker, and changes in biomarker.

### 2.5 Stage 5: Collating, summarizing, and reporting results

Once the eligible studies were extracted, we developed an analytical framework to collate, summarize, and synthesize the data. We made use of summary counts and tables to provide quantitative information on the body of research on blood biomarkers studied in the aforementioned population.

## 3 Results

### 3.1 Selection of included studies

**FIGURE 1:**
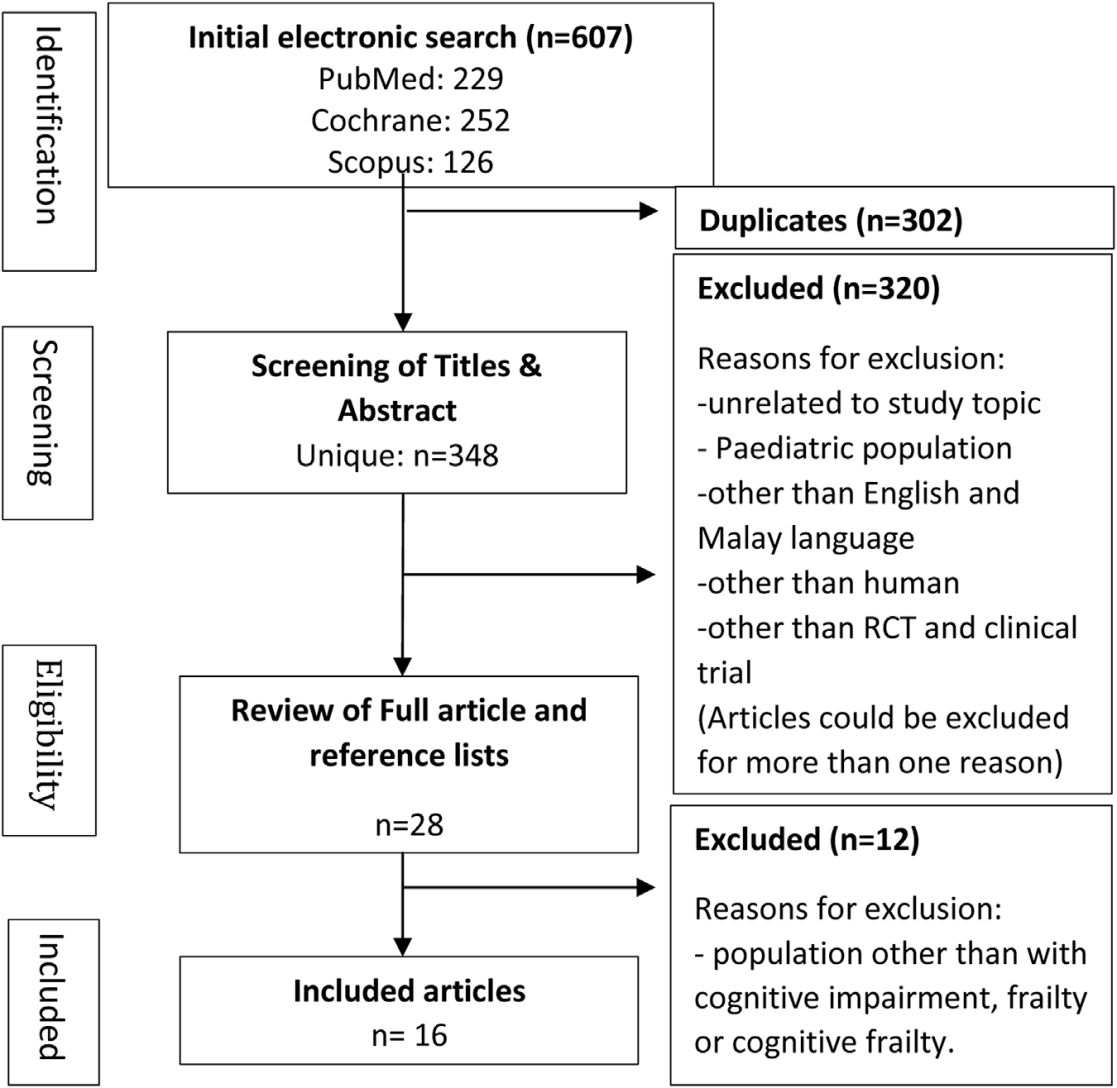
Syntax of literature search and selection process. PRISMA flow diagram: cardiovascular-health biomarkers in intervening older adults with cognitive frailty.

After screening for the keywords, a total of 607 articles were gathered from the three selected databases. Upon duplicate removal, 348 articles were screened for titles and abstracts. Following that, 28 articles were selected for full-text screening. Finally, a total of 16 articles were selected for data extraction (Figure 1).

### 3.2 General characteristics of included studies

All included studies were randomized controlled trials, while the intervention period varied from 12 to 96 weeks. These studies were published between 1999 and April 2022, and included both sexes and impairments, including frailty (*n* = 7) and cognitive impairment (*n* = 9), as depicted in Table 2.

**TABLE 2:** General characteristic of included studies.

Study	Age, year	Country	Sex	BMI, kg/m^2^	Duration, week	Impairment
Koblinsky et al. (2022)	60–85	Canada	Both	29.0 (5.0)–30.1 (5.1)	24	SCD or MCI
Roschel et al. (2021)	≥65	Brazil	Both	27.5 (5.2)–29.1 (3.9)	16	Frail (pre-frail and frail elderly)
Caldo-Silva et al. (2021)	≥75	Portugal		25.8 (3.1)–30.2 (3.7)	40	Frailty
Stephen et al. (2021)	60–70	Finland	Both	25.80 (3.30)–25.80 (3.30)	96	Cognitive impairment
Furtado et al. (2020)	≥75	Portugal	Both	28.19 (5.71)	28	Frailty
Sadjapong et al. (2020)	≥65	Thailand	Both	21.28 (0.69)–21.37 (0.68)	12	Frailty
Smith et al. (2020)	≥55	Unites States	Both	32.2 (4.7)–32.9 (3.6)	24	Cognitive impairment
Barha et al. (2019)	≥55	Canada	Both	25.78 (3.80)–26.64 (3.69)	48	Mild Vascular Cognitive Impairment
Tsai et al. (2019)	60–85	Taiwan	Both	23.36 ± 2.77–24.40 ± 3.08	16	aMCI
Ferreira et al. (2018)	≥60	Brazil	Both	26.4–26.8	12	Frailty
Tsai et al. (2018)	60–85	Taiwan	Both	23.83 ± 3.20– 24.48 ± 3.19	1 (acute)	MCI
Sungkarat et al. (2017)	≥60	Thailand	Both	23.6 (3.1)–23.9 (3.8)	15	MCI
Köbe et al. (2016)	60–80	Germany	Both	24.3 ± 0.8–25.4 ± 3.5	24	MCI
Kim et al. (2015)	≥75	Japan	Women	NR (not reported)	12	Frailty
Baker et al. (2010)	55–85	United States	Both	28.0 (5.5)–29.8(5.3)	24	MCI
De Jong et al. (1999)	≥70	Dutch	Both	≤25	17	Frailty

SCD, subjective cognitive decline; MCI, mild cognitive impairment; aMCI, amnestic mild cognitive impairment.

Out of 16 studies, there were five single-component intervention (exercise alone) studies that employed single type of exercise in an arm (aerobics, resistance, tai chi, and HIIT). Another three studies combined more than one type of exercises in an arm [aerobics + resistance (*n* = 1) and aerobics + resistance + balance (*n* = 2)]. There were a total of eight multicomponent intervention studies [exercise + diet (*n* = 3), exercise + supplementation (*n* = 4), and exercise + diet + vascular management + cognitive training (*n* = 1)].

### 3.3 Common type of biomarkers studied

In view of cardiovascular health-related biomarkers in response to exercise, most of the articles included inflammatory (*n* = 9), followed by glucose homeostasis (*n* = 9), lipid profile markers (*n* = 5), and hemostatic factors (*n* = 1) (Table 3).

**TABLE 3:** Cardiovascular-health related blood parameters and response to exercise intervention.

Study	Exercise type, frequency	n_T_/n_C_	Marker					Changes in markers
			Adipokine/inflammatory	Glucose homeostasis	Lipid profile	Hemostatic/thrombotic	Others	
Koblinsky et al. (2022)	Diet + Ex (AE + RE), HE + AE + RE/1 + 2 or 3 days/week	7/7	NA	HbA1c (Diet: 6.00 ± 0.29%–5.52 ± 0.18% HE + Ex: 5.76 ± 0.57%–5.71 ± 0.57%)	NA	NA	NA	HbA1c (−)
Roschel et al. (2021)	1) Leucine supplementation vs. placebo + Ex (RE), 2) whey vs soy supplementation vs placebo Ex (RE), 3) creatine vs whey vs creatine plus whey supplementation vs placebo + Ex (RE), and 4) whey supplementation vs placebo in women vs men + Ex (RE)/2 days/16 weeks	1) 22/22	NA	Placebo + RE:	Placebo + Resistance exercise:	NA	NA	1. IGF-1: (+)
		2) 22/22/22		1. IGF-1: 131.43–136.31	1. Cholesterol: 207.14–209.56			2. Glucose: (+)
		3) 22/22/22/22		2. Glucose: 95.98–96.76	2. HDL: 60.07–66.69			3. HbA1c: (+)
		4) 22/22/23/23		3. HbA1c: 5.72–5.72	3. LDL: 122.26–123.51			4. Insulin: (-)
				4. Insulin: 11.97–11.53	4. VLDL: 24.30–24.89			5. Cholesterol: (+)
					5. TG: 120.73–124.50			6. HDL: (+)
								7. LDL: (+)
								8. VLDL: (+)
								9. Triglycerides: (+)
Caldo-Silva et al. (2021)	ME (AR + R + B) + BCAAs, ME, BCAAs, control/2 days/week	8/7/7/13	1. IL-10: 8.68–10.53 2. TNF-α: 41.78–54.05	NA	NA	NA	1. Total albumin concentrations: 3.73–2.96	1. IL-10: (+)
			3. TNF-α/IL-10 ratio: 4.4–5.7					2. TNF-α: (+)
			4. MPO: 5,935.71–4,512.34					3. TNF-α/IL-10 ratio: (+)
								4. MPO: (−)
Stephen et al. (2021)FINGERS	Four-domain intervention-diet, exercise (AE + RE + BE), cognitive training, and vascular risk management, regular health advice/1–5x/week	59/53	NA	NA	1. TC 5.07 ± 1.03–4.76 ± 0.93	NA	NA	TC: (−)

Furtado et al. (2020)	CSE (AE + RE) and CME (RE), control non/2-3 days/week	21/20/19	1. CME: TNF-α to IL-10 ratio [9.30 (7.76)–6.46 (8.98)]	NA	NA	NA	NA	1. CME: TNF-α to IL-10 ratio (−)
			2. CME: IL-10 [13.91 (11.09)–18.38 (12.46)]					2. CME: IL-10 (+)
			CSE: IL-10 [17.63 (11.28)–21.55 (10.29)]					3. CSE: IL-10 (+)
			3. CRP (pg/mL) (=)					
			4. IFN-γ (−)					
Sadjapong et al. (2020)	MCEP (AE + RE + BE), 60 min, 3 days/week, 12 weeks	32/32	1. MCEP: CRP* (3.83–2.49)	NA	NA	NA	NA	IL-6*: (−) and CRP*: (−)
			2. MCEP: IL-6* (10.15–8.16)					
Smith et al. (2020)	AE + DASH and DASH, AE, HE	35/34/30/31	AE + DASH: -leptin: −10,633 (−15952, −5,314)	AE + DASH: -HOMA-IR: −0.6 (−1.1, −0.1)	NA	NA	AE + DASH: -neurotrophin z-score: –0.12 (–0.4, 0.16)	1. Leptin: (−)
ENLIGHTEN			Inflammation z-score*: −0.02 (−0.2, 0.2)	IGF-1: 0.5 (−2.9, 3.9)			AE:-neurotrophin z-score: 0.12 (–0.2, 0.4)	2. HOMA-IR: (−) for all, except HE
			AE: leptin: −3,245 (−8,857, 2,367)	AE:-HOMA-IR: −0.1 (−0.7, 0.5)				3. IGF-1: (+)
			Inflammation z-score*: −0.02 (−0.3, 0.2)	IGF-1: 2.2 (−1.4, 5.7)				4. Inflammation z-score (−)
								5. Neurotrophin z-score: (−) for AE + DASH and HE, (+) for DASH and AE
Barha et al. (2019)	AE, monthly HE/3 days/week	22/23	NA	NA	NA	NA	S100B (−3.096 pg/ml ± 2.388)	S100B (−)
Tsai et al. (2019)	AE and RE, Control/3 days/week	22/22/22	AE and RE: leptin (=)	AE: insulin (−)	NA	NA	AE and RE: VEGF (=),	1) AE and RE: leptin (=), VEGF (=), FGF-2 (=), IL-1β(=), IL-6 (=), and IL-8 (=)
			AE and RE: IL-1β (=),	RE: IGF-1 (+)			AE and RE: FGF-2 (=),	2) AE: peripheral serum BDNF level (+), insulin (−), TNF-α(−), and IL-15 (−) 3) RE: IGF-1 l (+) and IL-15 (−)
			AE and RE: IL-6 (=)				AE: peripheral serum BDNF level (+)	
			AE and RE: IL-8 (=)					
			AE: TNF-α (−)					
			AE and RE: IL-15 (−)					
Ferreira et al. (2018)	MC, usual activity/40 m, 3 days/week	13/24	MC: hsCRP*: 0.68 (0.46; 0.90)–2.41 (1.48; 3.35)	MC:-Glucose*: 100.9 (86.9; 115.0)–95.7 (88.3; 103.0),	MC:-TG*: 151.7 (119.1; 184.3) to 105.7 (83.7; 127.6),	NA	MC:- Vitamin D3*: 21.7 (17.3; 26.2)–26.0 (22.6; 29.5),	MC:-hsCRP*: (+), IL-6: (+), IL-10: (+), IL-1a: (+), and IL-1RAcP: (−)
			IL-6: 14.0–17.1 (14.39; 19.86)	Insulin*: 14.9 (10.1; 19.7)–11.3 (6.1; 16.4)	HDL: 48.6 (41.5; 55.7) to 49.1 (44.4; 53.8), TC*: 168.5 (144.9; 192.0) to 148.0 (132.9; 163.2), and			Glucose*: (−) and Insulin*: (−)
			IL-10: 13.0–15.6 (11.4; 19.7)		LDL: 87.9 (72.8; 103.1) to 83.5 (72.0; 95.0),			TG*: (−), HDL: (+), and TC*: (−)
			IL-1a: 14.6 (10.7; 18.4)–16.0 (14.0; 18.0)					LDL: (−)
			IL-1RAcP: 18.9 (12.9; 25.0)–16.1 (10.6; 21.6)					
Tsai et al. (2018)	AE and RE, control/40 m	25/21/20	NA	IGF-1: a) AE: +* then -b) RE: +* then −*	NA	NA	BDNF-higher in AE than control	IGF-1 (+) to (−)
							VEGF- AE*: (+)	BDNF level (+)
							FGF2 (=)	
Sungkarat et al. (2018)	Tai chi, control/3 days/week	33/33	Tai chi: TNF-α: 8.8 (4.7–33.0)–8.4 (4.5–31.6)	NA	NA	NA	BDNF*: 162.3 (22.6–721.0)–314.4 (30.3–693.3)	1. TNF-α: (−)
								2. BDNF: (+)
Köbe et al. (2016)	Group 1: Omega-3 FA + AE + cognitive stimulation (AKTIVA) and Group 2: Omega-3 FA + non-aerobic exercise/45 mins, 2 days/week	21/14	Group 1: Leptin: 4.0 ± 2.2–3.3 ± 2.8	Group 1: HbA1c: 6.0 ± 0.4–6.0 ± 0.5	Group 1: TG *: 110.3 ± 47.8 to 83.0 ± 30.5,	Group 1: Homocysteine*: 15.9 ± 7.0–12.5 ± 3.8	Group 1: BDNF: 4,670.8 ± 1,538.9–3,781.2 ± 1,136.9	Group 1: 1. Leptin: (−)
				IGF-1: 138.5 ± 28.6–134.7 ± 34.004	TC: 210.2 ± 27.4 to 208.9 ± 41.1			2. HbA1c: (−)
					LDL-to-HDL ratio:			3. IGF-1: (−)
					2.1 ± 0.7 to 2.0 ± 0.8			4. TG*: (−),
								5. TC: (−)
								6. LDL-to-HDL ratio: (−)
								7. Homocysteine: (−)
								8. BDNF: (−)
Kim et al. (2015)	Ex (RE + BDD)1 + MFGM, Ex1 + placebo, and MFGM, placebo/2 days/week	33/33/32/33	NA	(IGFBP3/IGF-1) x 100*: 4.18 ± 1.46 to 4.90 ± 2.46	NA	NA	Serum BDNF: 6.37 ± 1.44 to 7.07	IGFBP3/(IGF)-1 ratio*: (+)
							± 1.01	Serum BDNF: (+)
Baker et al. (2010)	AE (high-intensity aerobic exercise), SE/4 days/week	19/10	NA	1. HOMA: AE (+) women and men	1. TBF: (−) Men and women in AE more than SE	NA	BDNF:	AE improves:
				2. Glucose disposal*: AE (+) women	2. TC*: (−) AE and (+) SE		AE- (−) men, (+) women	1. HOMA: (+) women and men
				3. Insulin: AE (+) women	3. LDL: (=)			2. Glucose disposal*: (+) women
				4. IGF-I: AE (+) men	4. HDL: (=)			3. Insulin: (+) women
					5. TG: (=)			4. IGF-I: (+) men
								5. TBF: (−) women and men
								6. TC*: (−) women and men
								BDNF: (+) women
De Jong et al. (1999)	Control, Ex, nutrition + Ex (AE + RE + CE.), and nutrition/2 days/week	34/35/39/37	1. CRP: –0.1 ± 1.9 (change in the Ex group)	NA	NA	NA	NA	CRP: (−)

n_T,_ sample size of the treatment group; n_C,_ sample size of the control group; MPO, myeloperoxidase; IL-6, interleukin 6, BDNF, brain-derived neurotrophic factor; CRP, C-reactive protein.

Ex, exercise; MCT, multicomponent training; MCEP, multicomponent exercise program (aerobic exercise, resistance exercise, and balance exercise); ME, multicomponent exercise; CME, chair multimodal exercise; CSE, chair elastic band muscle strength exercise, BCAAs, branched-chain amino acid supplementation; AE, aerobic exercise; RE, resistance exercise; BE, balance exercise; CE, coordination exercise; SE, stretching exercise; DASH, Dietary Approaches to Stop Hypertension (DASH); HE, health education; (+), increase; (−), decrease; (=), no changes.

aMCI, amnestic mild cognitive impairment, *, *p*-value < 0.05.

SMP30, regucalcin or senescence marker protein-30; HbA1c, glycated hemoglobin; TNF-α, tumor necrosis factor-alpha; ; IL-8, interleukin 8; IL-10, interleukin 10; IL-1β, interleukin 1β; IL-1RAcP, interleukin-1 receptor accessory protein;, VEGF, vascular endothelial growth factor; IGF-1, insulin-like growth factor 1; FGF-2, fibroblast growth factor-2; S100B, S100 calcium-binding protein B; IFN-γ, interferon-gamma; TC, total cholesterol; LDL, low-density lipoprotein; HDL, high-density lipoprotein; TG, triglycerides; BDNF, brain-derived neurotrophic factor; VLDL, very low-density lipoprotein; HOMA, homeostasis model assessment of insulin resistance.

Inflammatory biomarkers studied were IL-6, IL-8, IL-10, IL-15, IL-1β, TNF-α, IFN-γ, CRP, and leptin. Most studies reported that exercise intervention reduced pro-inflammatory cytokines, including IL-6 (Sadjapong et al., 2020), TNF-α (Furtado et al., 2020; Sungkarat et al., 2018; Tsai et al., 2019), and IL-15 (Tsai et al., 2019). However, a study by Tsai et al. (2019) showed no changes in other pro-inflammatory cytokines, namely, IL-6, IL-8, IL-1β, and pro-inflammatory regulate—leptin. Interestingly, pro-inflammatory regulate—leptin was shown to be reduced with both aerobic exercise intervention studies (Smith et al., 2020; Köbe et al., 2016). C-reactive protein (CRP) was shown to be significantly reduced with exercise intervention in the studies by Ferreira et al. (2018) and De Jong et al. (1999), but no changes were reported in the study by Furtado et al. (2020). IL-6, on the other hand, was shown to be reduced in the study by Sadjapong et al. (2020) and increased in the study by Ferreira et al. (2018) and no changes were reported in the study by Tsai et al. (2019).

Anti-inflammatory markers, such as IFN-γ and IL-10, have also been studied. IFN-γ and IL-10 improved with resisted chair-based exercise interventions (Furtado et al., 2020), and IL-10 improved with multicomponent exercise (Ferreira et al., 2018; Caldo-Silva et al., 2021). Previous studies indicated that older adults with cognitive impairment and/or frailty had higher inflammatory biomarkers (Langmann et al., 2018; Pothier et al., 2022). Exercise interventions have been consistent in reducing pro-inflammatory biomarkers among healthy older adults (Vanhees et al., 2012; Zheng et al., 2019).

The second commonly reported markers were glucose homeostasis markers, consisting of HbA1c, IGF-1, glucose, insulin, and HOMA-IR. Exercise improved glucose homeostasis markers *via* reduced HbA1c (Koblinsky et al., 2022; Köbe et al., 2016), increased IGF-1 (Roschel et al., 2021; Smith et al., 2020; Tsai et al., 2019; Baker et al., 2010), reduced insulin (Roschel et al., 2021; Tsai et al., 2019; Ferreira et al., 2018), reduced HOMA-IR (Smith et al., 2020), and reduced glucose (Ferreira et al., 2018). Physical activities are effective in hypoglycemic control to enhance cognitive function and to reduce the risk of dementia (Wang et al., 2021).

Lipid profiles, namely, total cholesterol (*n* = 5), triglycerides (*n* = 4), HDL (*n* = 3), LDL (*n* = 3), and total body fat (*n* = 1) were reported in five studies. Beneficial effects of exercise on the lipid profile were observed by the decrement in TC (Baker et al., 2010; Köbe et al., 2016; Ferreira et al., 2018; Stephen et al., 2021), TG (Köbe et al., 2016; Ferreira et al., 2018), and LDL (Ferreira et al., 2018). Exercise, especially aerobic exercise, has been reported effective in improving the lipid profile among healthy adults and even Alzheimer population (Kelley et al., 2005; Jensen et al., 2020). Lipid contribution to Alzheimer’s disease can be explained by the involvement of apolipoprotein, which is a key lipid-transport protein in high-risk late-onset AD.

Only one study by Köbe et al., 2016 reported on the hemostatic factor, homocysteine. This study indicated a reduction in homocysteine with exercise intervention in combination with other interventions.

### 3.4 Changes in cardiovascular-health biomarkers with exercise intervention

It would have been beneficial to find a specific type of exercise that is superior in improving cardiovascular health biomarkers among older adults with cognitive impairment, frailty, or cognitive frailty. First and foremost, 50% (*n* = 8) of the studies involved multicomponent exercise (Koblinsky et al., 2022; Caldo-Silva et al., 2021; Stephen et al., 2021; Sadjapong et al., 2020; Ferreira et al., 2018; Tsai et al., 2018; De Jong et al., 1999; Sungkarat et al., 2018; Guilherme et al., 2020; Kim et al., 2020).

Seven out of the eight studies included aerobic exercise on its own (Baker et al., 2010; Köbe et al., 2016; Tsai et al., 2018; Smith et al., 2020) and as part of multicomponent exercise (Ferreira et al., 2018; Tsai et al., 2019; Koblinsky et al., 2022). They showed improvement in glucose metabolism *via* various parameters, including Hba1c, HOMA-IR, IGF-1, and glucose disposal *via* metabolic clamp. Only one study carried out a comparison between aerobic and resistance exercises, and the results showed that aerobic exercise reduced the insulin level, while resistance exercise increased the IGF-1 level (Tsai et al., 2019). Baker et al. (2010) compared aerobic and stretching exercises among 33 older populations with MCI. These authors found that high-intensity aerobic exercise significantly improved glucose disposal during metabolic clamp, insulin sensitivity, and BDNF among women. Meanwhile, for men, aerobic exercise improved IGF-1.

Concerning body fat, the total body fat was reduced in both men and women in aerobic groups, while the total cholesterol significantly reduced with aerobic exercise (Baker et al., 2021) but increased in the stretching group (Baker et al., 2010). LDL was found to have an analogous pattern but not HDL and triglycerides.

In the four studies that have yielded improvements in the lipid profile, only two studies carried out aerobic exercise (Köbe et al., 2016; Baker et al., 2021). On the other hand, the other two aerobic exercises were performed in combination with the resistance and balance exercises (Stephen et al., 2021) or resistance exercise, mobility, and flexibility (Ferreira et al., 2018). With regard to single-component exercise, aerobic exercise among patients with MCI significantly reduced their homocysteine and triglyceride values (Köbe et al., 2016). Improvement trends in several parameters, namely, leptin, IGF-1, total cholesterol, LDL/HDL ratio, and BDNF level were also found. Second, in the study by Furtado et al. (2020), multimodal chair-based and chair-based exercises with the elastic band were compared. The results found that there was an increase in IL-10 and decrease in IFN.

## 4 Discussion

### 4.1 Key findings

In this scoping review, we mapped changes in cardiovascular health-related blood parameters among older adults with cognitive frailty in response to exercise on its own or as a component of the intervention. To the best of our knowledge, this is the first review covering the impact of exercise intervention on older adults with cognitive frailty. The overall results showed that the cardiovascular health-related biomarkers studied were mostly inflammatory, glucose-metabolism-related, and lipid profile markers.

Among all the inflammatory parameters, a reduction was observed in TNF-α (Tsai et al., 2019; Sungkarat et al., 2018; Furtado et al., 2020) and leptin (Köbe et al., 2016; Smith et al., 2020). The increase in TNF-α in the study by Caldo-Silva et al. (2021) could be due to the higher comorbidity index and lower cognitive profile. In addition, leptin decreased in response to aerobic exercise intervention (Köbe et al., 2016; Smith et al., 2020). Leptin stimulates fatty acid oxidation while decreasing incorporation of fatty acid into intramuscular lipid droplets. Ageing process can be described with low-grade chronic inflammation, leading to muscle loss. Previous studies have shown that multicomponent exercise is more effective in improving inflammatory markers (Sadjapong et al., 2020). Inflammatory markers, such as CRP, IL-6, and TNF-α, were reported as stable and least likely to be affected by exercise behavior (Che and Li, 2017). Cardiovascular biomarkers were also categorized based on inflammatory factors, functional metabolomics, and heart-specific proteome (Che and Li, 2017). However, the response to exercise on the stated biomarkers was scarce.

In view of the type of exercise that exerted anti-inflammatory effects, three studies using multicomponent exercise showed positive changes in anti-inflammatory biomarkers (Ferreira et al., 2018; Guilherme et al., 2020; Caldo-Silva et al., 2021). In a recent systematic review that determined the effectiveness of aerobic exercises on inflammatory markers in healthy middle-aged and older adults, positive changes in inflammatory markers were reported (Zheng et al., 2019).

In our present review, we found that multicomponent exercises led to positive changes in anti-inflammatory biomarkers among older adults with cognitive impairment and frailty. This could be due to the fact that multicomponent exercises are beneficial to addressing many associated issues related to both cognitive impairment and physical frailty, such as weakness, impaired postural stability, fear of falling, and quality of life. As a result, there is holistic improvement in physical and psychosocial health. This is further supported by evidence that multicomponent exercise interventions are beneficial to the prevention and reversal of cognitive impairment and/or frailty (Cadore et al., 2019; Sáez de Asteasu et al., 2019; Kim et al., 2020; Murukesu et al., 2020; Murukesu et al., 2021).

An increase in age is associated with glucose intolerance and insulin resistance. With skeletal muscle being the main target for glucose uptake, the structural and metabolic changes of this tissue associated with ageing has a significant role in pathogenesis of insulin resistance in the ageing population. Myriad benefits of aerobic exercise on glucose metabolism can be explained *via* improved skeletal muscle insulin signaling factor (AS160), GLUT4 protein content, and glycogen synthase and oxidative (PDH). These changes are similar to those by resistance exercise except that there is limited improvement in skeletal muscle GLUT4 and insulin-stimulated glycogen synthase pathways (Consitt et al., 2019).

A reduction in total cholesterol among participants in all four studies (Baker et al., 2010; Köbe et al., 2016; Roschel et al., 2021; Stephen et al., 2021) is also supported by the study by Da Silva et al. (2016). These can be explained by the increase in the activity of lipase lipoprotein and lecithin cholesterol acyltransferase and the reduced activity of hepatic lipase and cholesterol esterified transfer protein which reverses cholesterol transport (Lehmann et al., 2001; Da Silva et al., 2016).

### 4.2 Research Gaps

There is limited research regarding the effectiveness of exercise on cardiovascular health-related parameters among older adults with cognitive impairment or frailty, especially cognitive frailty. More information is required pertaining to this matter to assist in designing the best evidence-based exercise program in the prevention and management of cognitive frailty in older adults.

### 4.3 Study limitations

This review reported important findings. However, several limitations should be acknowledged. First, the quality appraisal of studies was not assessed. Second, three databases were accessed. Although it is adequate, using more databases may allow a better understanding.

## 5 Conclusion

In the present review, we found that exercise intervention yielded positive outcomes in cardiovascular health-related biomarkers, particularly pro-inflammatory markers, namely, IL-6, TNF-α, IL-15, leptin, and C-reactive protein and anti-inflammatory markers, namely, IFN-γ and IL-10. Similarly, glucose metabolism-related biomarkers, namely, IGF-1 and HbA1c, glucose, and insulin sensitivity were shown to be improved with exercise intervention. Most studies, which showed improved biomarker outcomes, were in response to multicomponent exercise (combination of aerobic, resistance, flexibility, and balance exercises) and only aerobic exercise.

## Data Availability

The original contributions presented in the study are included in the article/Supplementary Material; further inquiries can be directed to the corresponding author.
